# *Nearest-cell*: a fast and easy tool for locating crystal matches in the PDB

**DOI:** 10.1107/S0907444912040590

**Published:** 2012-11-09

**Authors:** V. Ramraj, G. Evans, J. M. Diprose, R. M. Esnouf

**Affiliations:** a The Division of Structural Biology, Wellcome Trust Centre for Human Genetics, University of Oxford, Oxford OX3 7BN, England; b Diamond Light Source, Harwell Science and Innovation Campus, Didcot OX11 0DE, England

**Keywords:** *Nearest-cell*, crystal matches, Protein Data Bank

## Abstract

When embarking upon X-ray diffraction data collection from a potentially novel macromolecular crystal form, it can be useful to ascertain whether the measured data reflect a crystal form that is already recorded in the Protein Data Bank and, if so, whether it is part of a large family of related structures. Providing such information to crystallographers conveniently and quickly, as soon as the first images have been recorded and the unit cell characterized at an X-ray beamline, has the potential to save time and effort as well as pointing to possible search models for molecular replacement. Given an input unit cell, and optionally a space group, *Nearest-cell* rapidly scans the Protein Data Bank and retrieves near-matches.

## Abstract 2

A fast and easy tool to locate unit-cell matches in the PDB is described.

## 1. Introduction

X-ray crystallography remains the primary method for the determination of the atomic structure of biological macromolecules. At the time of writing, more than 80 000 structures form the Protein Data Bank (PDB; http://www.wwpdb.org; Westbrook *et al.*, 2005 ▶), of which roughly 87% have been solved using X-ray crystallography.

The rate at which macromolecular crystallography (MX) data sets can now be measured at synchrotron-radiation facilities (Winter & McAuley, 2011 ▶) raises issues relating to the effective use of beamtime. Automated tools that allow synchrotron beamline users to be as efficient as possible are under continual development (Bahar *et al.*, 2006 ▶; Keegan & Winn, 2007 ▶; Panjikar *et al.*, 2009 ▶; Winter & McAuley, 2011 ▶). The tool described here, *Nearest-cell*, is a useful addition to this automation armoury.

Somewhat masked by the success of MX, numerous challenges remain in protein production, purification and crystallization. This is particularly the case for complexes comprising multiple protein subunits as well as membrane proteins, where there can be an elevated risk of purifying host-system expression byproducts along with the target of interest. Given the difficulties associated with crystallizing many of these ‘high-impact’ targets, it is often the case that the ‘impurity’ protein crystallizes more readily. A ready way of determining whether a crystal might arise from an impurity, such as *Nearest-cell*, is particularly useful in these situations.

*Nearest-cell* has been installed at the MX beamlines at the Diamond Light Source and uses output from automated data-analysis pipelines such as *fast_dp* (Winter & McAuley, 2011 ▶) to provide users with a putative list of similar unit cells (and hence, potentially, structures) in the PDB.

## 2. Experimental procedures

*Nearest-cell* depends on a custom set of software (a pipeline) designed to update an internal database. It was written to be executed weekly, coinciding with updates of the PDB.

### 2.1. Database pipeline

The pipeline is written in C++; it updates a database of key information (PDB ID, organism, experimental method, unit cell, space group, *R* factors) from PDB XML files and consists of software and a database that is used to store the necessary information for rapid retrieval by *Nearest-cell*. It uses part of the *PHENIX* software suite (Adams *et al.*, 2010 ▶), specifically *phenix.explore_metric_symmetry*, to pre-compute the reduced symmetry *P*1 cell for a given unit cell and space group. The pipeline parses the unit cell corresponding to space group *P*1 from the *phenix.explore_metric_symmetry* output and stores it in the database.

The pipeline runs automatically to coincide with the PDB update schedule and performs the following tasks.

(i) Synchronize a local PDB XML repository with the PDBe mirror.
(ii) Extract key information from new or changed PDB entries and add it to the database. Purge superseded entries.
(iii) Run *phenix.explore_metric_symmetry* on each updated PDB entry; store *P*1 cell in the database.
(iv) Generate flat file SEQRES and ATOM records for each updated PDB XML file.

#### 2.1.1. Auxiliary pipeline features

The pipeline also stores the number of space-group symmetry operators for each space group. While *PHENIX* can be invoked each time for this information as required, it is faster for *Nearest-cell* to retrieve this information from a database. These data are used by the family-clustering algorithm (described below). The pipeline also allows the manual curation of alternate space groups and indexing conventions that occasionally arise in the PDB.

The SEQRES records that are generated by the pipeline are simple *FASTA* format files containing descriptive headers and single-letter amino-acid sequences for each chain of a PDB entry. The single-letter sequence is derived from the three-letter amino-acid code in the PDB XML file. To account for nonstandard amino acids, the pipeline is able to call a JSON web service developed by the EBI for this specific purpose (personal communication with Jose Dana and Sameer Velankar of the EBI) to retrieve the appropriate standard amino acid for a given nonstandard input. For example, the amino acid selenomethionine, coded in a PDB record as ‘MSE’, is resolved by the JSON web service to ‘M’ (methionine). Once again, it is advantageous to retrieve all nonstandard amino-acid mappings in advance, since the JSON query is slower and relies on an external server. The pipeline has the capacity to pre-fetch and store all of these mappings to the database, although this feature need not be run weekly.

### 2.2. *Nearest-cell*

**Figure 1:**
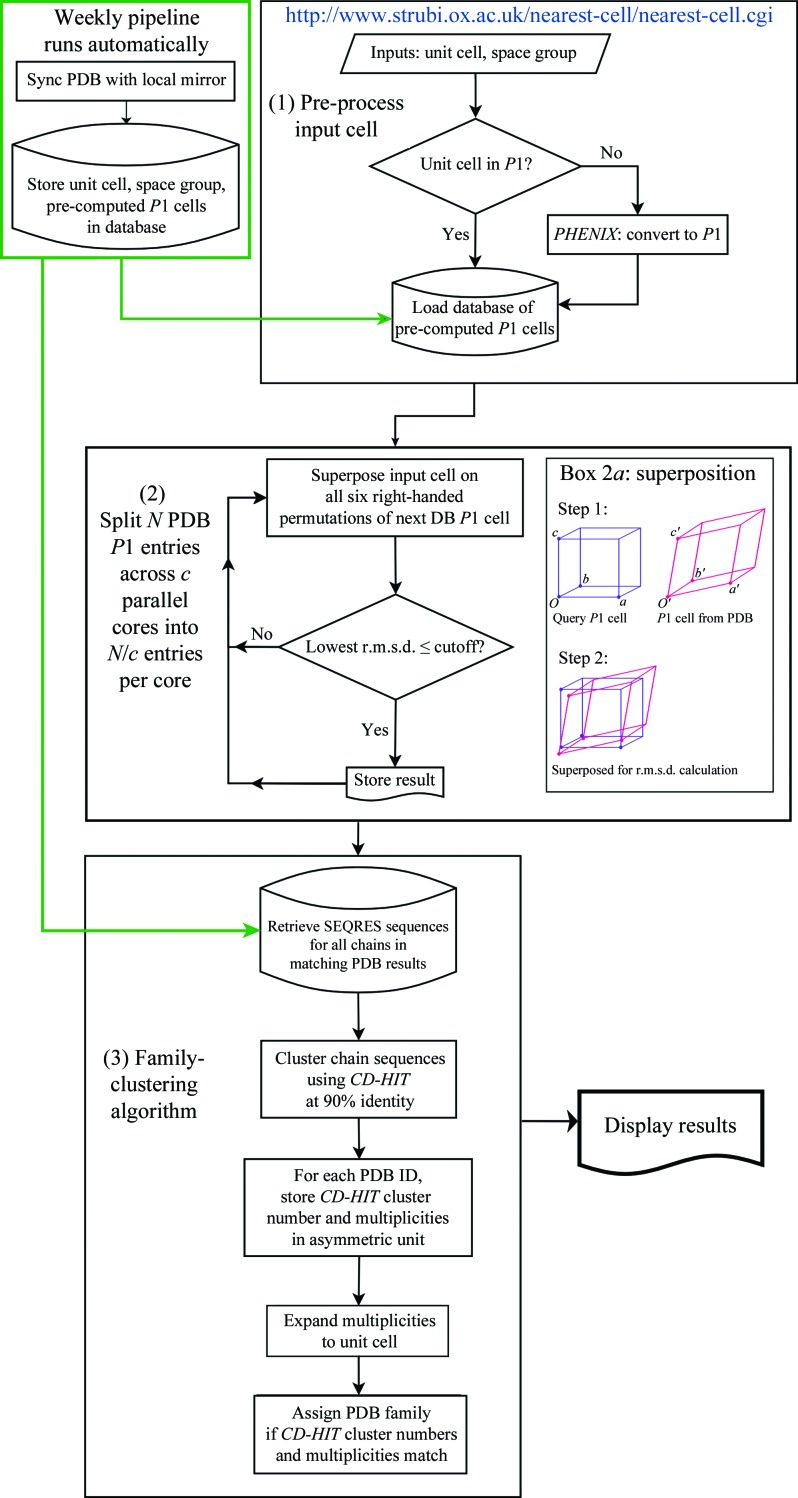
Schematic showing *Nearest-cell*’s logic. (1) The input cell is first converted to *P*1 if required. (2) It is then compared with every known *P*1 cell in the PDB using *MATFIT* (McLachlan, 1972 ▶; Kabsch, 1976 ▶, 1978 ▶); the schematic in box 2a shows an example superposition with one permutation of the database *P*1 cell (*O*′ superposed on *O*, *A*′ on *A*, *B*′ on *B* and *C*′ on *C*). If the lowest r.m.s. difference of all six superpositions is less than the specified cutoff (see §[Sec sec2.2.1]2.2.1), the database cell qualifies as a positive match. (3) The family-clustering algorithm clusters PDB entries into families of sequence similarity. Results are then displayed to the user with each family represented by the PDB entry with the smallest r.m.s. difference from the input. Families can be expanded to show all hits, as shown in Fig. 2 ▶.

*Nearest-cell* is a multi-process capable command-line driven C++ application with a Python web service front end. Fig. 1 ▶ describes the logic underpinning *Nearest-cell*. When invoked, it calls several external applications.

(i) *phenix.explore_metric_symmetry* for reducing the query unit cell to a *P*1 unit cell.
(ii) *MATFIT*, a superposition subroutine (McLachlan, 1972 ▶; Kabsch, 1976 ▶, 1978 ▶) described in §[Sec sec2.2.1]2.2.1.
(iii) *CD-HIT* (Li & Godzik, 2006 ▶), a sequence-clustering method, as part of the family-clustering algorithm (§[Sec sec2.2.2]2.2.2).

#### 2.2.1. *MATFIT*

This is a Fortran subroutine that calculates the rotation matrix and translation vector for the best superposition of two sets of atomic position vectors (McLachlan, 1972 ▶; Kabsch, 1976 ▶, 1978 ▶) and returns an r.m.s. difference. When *Nearest-cell* compares the input *P*1 cell against a pre-computed PDB *P*1 cell in its database, it tests all six valid right-handed combinations of axes (since proteins are enantiomorphic), running *MATFIT* each time and choosing the smallest r.m.s. difference of the six. If this lowest r.m.s. difference is within a cutoff (either specified on the command line or, by default, set to the larger of 2.5 Å or 1% of the sum of the longest and the shortest unit-cell dimensions), the PDB cell qualifies as a positive match. Box 2a in Fig. 1 ▶ shows one such comparison between the query *P*1 cell and a database *P*1 cell.

#### 2.2.2. *CD-HIT*

This program (Li & Godzik, 2006 ▶) groups amino-acid sequences into clusters at a desired level of sequence identity (set to 90% of the length of the shortest sequence by default). Each cluster is described by a representative sequence. It is used here as a preliminary clustering step for the family-clustering algorithm described below.

### 2.3. Family-clustering algorithm

**Figure 2:**
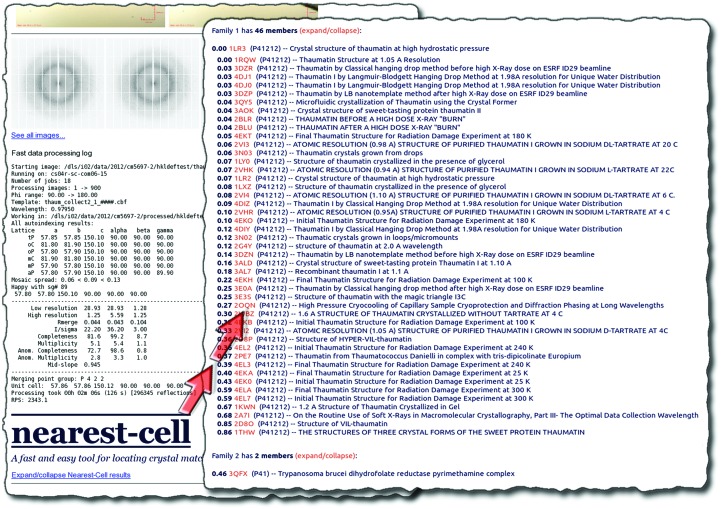
Typical output from *Nearest-cell*, shown as part of Diamond’s *fast_dp* report for a thaumatin unit cell. The results are appended to the end of a *fast_dp* run. Family 1 contained 46 thaumatin unit cells clustered together, showing the effectiveness of the family-clustering algorithm for reducing the number of results displayed to the user (inset). Note that this family contains two exact matches (r.m.s. difference = 0.00 Å).

This algorithm was developed to exploit similarity at the sequence level to usefully group matches at the PDB-record level, which can contain different numbers of chains and/or belong to different space groups. This allows *Nearest-cell* to substantially reduce the output. The problem is evident for an input cell matching that of horse heart myoglobin (PDB entry 3vau; Yi & Richter-Addo, 2012 ▶), for instance, which produces 126 hits when run through *Nearest-cell*. Since most of the hits are from the same family (myoglobin), the family-clustering algorithm reduces the output to representative PDB IDs for each of just five families. The output from a more typical query is shown in Fig. 2 ▶.

The basic logic of the algorithm is shown in step 3 of Fig. 1 ▶. All sequences from all PDB entries with matching *P*1 cells are grouped into clusters using *CD-HIT*. The contents of the asymmetric unit for each PDB entry can then be described by how many examples of each cluster it contains. This can then be expanded to describe the *P*1 cell by multiplying by the number of symmetry operators for the space group. Finally, a pair of PDB IDs are clustered together into the same family only if the *CD-HIT* cluster numbers and multiplicities match.

## 3. Results and discussion

*Nearest-cell* is currently available for public use through the web service located at http://www.strubi.ox.ac.uk/nearest-cell/nearest-cell.cgi.

The web service takes a unit cell as required input. Space group is optional, and if not provided is assumed to be *P*1. In parallel computation mode, using two cores on a modern computer, computation takes just under 1 s. Across 24 cores, this computation time is reduced to 0.3 s. The entire web-service request from start to finish takes about 5 s if the space group is *P*1 and about 10 s otherwise (the overhead of invoking *PHENIX* to reduce the unit cell to *P*1 using *phenix.explore_metric_symmetry*). Note that space groups need to be in *PHENIX*-accepted format (Adams *et al.*, 2010 ▶). The CGI script can also be invoked using a GET request with the parameters in the URL; for example, http://www.strubi.ox.ac.uk/nearest-cell/nearest-cell.cgi?unit-cell=24,24,24,90,90,90&space-group=R3:R. In this way, URLs can be generated programmatically as part of other pipelines. This is especially useful for facilities such as the Diamond Light Source, where *Nearest-cell* has been integrated into internal pipelines such as *fast_dp* (Fig. 2 ▶; Winter & McAuley, 2011 ▶).

## 4. Conclusion

The design decision for *Nearest-cell* was to base match solely on the unit-cell dimensions rather than attempting to match (low-resolution) structure factors. Although our approach is less selective and gives more false positives, it allows *Nearest-cell* to be run more rapidly and directly after unit-cell characterization, thereby informing effective use of beamtime. While current PDB search tools do allow a search of unit-cell dimensions within given tolerances, this does not provide the comprehensive matching provided by *Nearest-cell*. The more rigorous approach of matching structure factors is embodied within molecular-replacement strategies such as *BALBES* (Long *et al.*, 2008 ▶).
